# Farm Management in Organic and Conventional Dairy Production Systems Based on Pasture in Southern Brazil and Its Consequences on Production and Milk Quality

**DOI:** 10.3390/ani5030367

**Published:** 2015-07-09

**Authors:** Shirley Kuhnen, Rudinei Butka Stibuski, Luciana Aparecida Honorato, Luiz Carlos Pinheiro Machado Filho

**Affiliations:** 1 Department of Zootechny and Rural Development, Federal University of Santa Catarina, Florianópolis 88034-001, Brazil; E-Mail: shirley.kuhnen@ufsc.br; 2 Graduate Program in Agroecosystems, Federal University of Santa Catarina, Florianópolis 88034-001, Brazil; E-Mails: rudistibuski@hotmail.com (R.B.S.); honorato.luciana@ufsc.br (L.A.H.)

**Keywords:** organic milk, pasture-based dairy production, sustainable agriculture, food security

## Abstract

**Simple Summary:**

This study provides the characteristics of the conventional high input (C-HI), conventional low input (C-LI), and organic low input (O-LI) pasture-based production systems used in Southern Brazil, and its consequences on production and milk quality. C-HI farms had larger farms and herds, annual pasture with higher inputs and milk yield, whereas O-LI had smaller farms and herds, perennial pastures with lowest input and milk yields; C-LI was in between. O-LI farms may contribute to eco-system services, but low milk yield is a major concern. Hygienic and microbiological milk quality was poor for all farms and needs to be improved.

**Abstract:**

Pasture-based dairy production is used widely on family dairy farms in Southern Brazil. This study investigates conventional high input (C-HI), conventional low input (C-LI), and organic low input (O-LI) pasture-based systems and their effects on quantity and quality of the milk produced. We conducted technical site visits and interviews monthly over one year on 24 family farms (n = 8 per type). C-HI farms had the greatest total area (28.9 ha), greatest percentage of area with annual pasture (38.7%), largest number of lactating animals (26.2) and greatest milk yield per cow (22.8 kg·day^−1^). O-LI farms had the largest perennial pasture area (52.3%), with the greatest botanical richness during all seasons. Area of perennial pasture was positively correlated with number of species consumed by the animals (R^2^ = 0.74). Milk from O-LI farms had higher levels of fat and total solids only during the winter. Hygienic and microbiological quality of the milk was poor for all farms and need to be improved. C-HI farms had high milk yield related to high input, C-LI had intermediate characteristics and O-LI utilized a year round perennial pasture as a strategy to diminish the use of supplements in animal diets, which is an important aspect in ensuring production sustainability.

## 1. Introduction

Milk is the top agriculture commodity in monetary value in the world, and Brazil is the world’s fourth largest fresh cow milk producer [1]. Within Brazil, Western Santa Catarina State, Southern Brazil, is the third largest and fastest growing milk-producing region [2]. On its way to becoming a major player in milk exports, the region is characterized by diversity in dairy production systems and is dominated by family farming (80% of farms) [3]. The region has a mild, subtropical humid climate, without a dry season and with temperate summers, allowing year round pasture-based management. These pasture systems can consist of either extensive or rotational grazing, and animals may be supplied with different amounts of supplements (concentrate and silage). In semi-intensive systems, cows are fed with large amounts of supplements. On the other hand, free-stall confinement is rare in the region [4].

World organic food production is growing at a rate of 10%–15% annually [5]. The growth of the organic market is likely associated with consumers’ concerns related to human health risks (e.g., degenerative diseases) caused by the use of pesticides, antibiotics, hormones and heavy metals in agriculture [6]. Furthermore, environmental impact and animal and human welfare are important considerations in organic farming practices and, therefore, organic production has been adopted by many family dairy farms globally. In Western Santa Catarina State, dairy farmers have been converting to organic production due to the assumed lower production costs while maintaining high levels of performance [4,7]. It is important to note that, regardless of the management system used, maximizing the use of forage to feed ruminants is essential in improving eco-efficiency [8]. Concentrate use increases with dairy farm intensification [8,9]. However, recommendations to improve the environmental impact of dairy farms include reducing the use of concentrates per kg of milk so as to improve the nutrient flow of the farm [9].

Comparative studies between conventional and organic pasture-based production systems are rare in the literature. The majority of studies conducted in the USA and Europe compare organic with free-stall farming, which are different from the dairy systems used in Brazil (e.g., see: [10,11]). Nevertheless, some studies have found differences between systems in relation to feed management, the use of forage species [12,13] and eco-efficiency [8]. Animals that receive a diet based on pasture, compared to a diet based on preserved forage and concentrate supplements, produce milk with greater concentrations of chemical components that are beneficial for human health, including conjugated linoleic acid, carotenoids, lipid-soluble vitamins A and E and antioxidants [10,11]. For consumers, knowledge about differences in milk quality is important for decision making related to food choice; for farmers, knowing what causes differences in food quality and production performance is important information for decision making related to production and management.

Recently, dairy industries have increased their presence in the study region, increasing milk processing capacity [4] and, as a consequence, putting pressure on dairy farmers to increase their production, which entails use of high-yield dairy breeds and a considerable input of feed grain that are known to rapidly increase productivity [14]. Despite increasing production, intensification may also increase costs and have significant environmental impacts [15]. A study providing a detailed assessment of the characteristics of pasture-based production systems used in a typical and growing dairy region may provide knowledge that can be used to inform future applied studies or decision making related to milk quality. Our field study seeks to describe pasture-based dairy production systems used in Southern Brazil and to assess the possible effects of management on diet composition, milk production, composition and quality. To our knowledge, this is the first study comparing production traits and milk quality between conventional and organic pasture-based production systems.

## 2. Materials and Methods

This study was conducted in Santa Catarina State, Southern Brazil, between May 2011 and April 2012. Twenty-four family farms were included in the analysis, and each farm was visited monthly. Based on earlier studies [4,7,16] and information gathered from technical assistants in the region, only pasture-based farms were included. We defined three categories of dairy production systems based on the typical management from that region: Conventional High input Pasture-based (C-HI), Conventional Low input Pasture-based (C-HI), and Organic Low input Pasture-based (O-LI). Eight farms per management type were included in the study. C-HI farms have a high intake of concentrates and preserved forage year round (>10 kg DM/cow/day); C-LI farms have a low intake of concentrates and preserved forage (<10 kg DM/cow/day); and O-LI farms are low input pasture-based dairy farms that are certified to be organic by the Ecovida Network in accordance with Brazilian Legislation [17].

All farms included in the study were located in Santa Catarina, with similar soil and climate characteristics. The climate is humid subtropical (Cfb in the Köppen classification) with an annual rainfall between 1900 and 2200 mm and a mean air temperature of 18 to 20 °C [18]. The farmers have similar ethnic background and use similar agricultural techniques. We used semi-structured interviews to collect data on the following variables on the first visit: total farm area, land use, area for cultivating perennial and annual pasture. During each monthly visit we collected data on breed of animals, number of lactating animals, monthly herd milk yield (obtained from sales records) and diet composition. In assessing nutritional management, we recorded the production of silage (tons/year) and estimated the daily consumption of silage (kg/cow), and daily consumption of concentrate (obtained from sale records) (kg/cow). For the calculation of silage and concentrate dry matter intake, the dry matter concentration in silage and concentrate samples were analyzed according to Van Soest [19]. The silage and concentrate dry matter intake was estimated based on the information given by the producers and dry matter determined from collected samples. We determined the forage species consumed by the animals using the methodology described by Euclides *et al.* [20], which consists of simulated grazing after observing the feeding behavior of animals for 30 min. The sampling of plant species consumed by the animals during observation was performed at each farm and during each season throughout the year.

Due to differences in the breeds used at each farm, and as recommended by other authors [4,7], milk samples for chemical analysis were obtained from the milking of three pre-selected animals based on lactation period (between the 4th and 6th month), phenotype and similar genetic composition (3/4 Holstein and 1/4 Jersey). The total milking of the three selected cows was mixed and a sample was collected for chemical analysis. Further, milk samples from the bulk tank of each farm were collected for the analysis of somatic cell count and bacteria count (SCC and BMBC). The procedure described above was performed twice in each season, on alternate days, and all samples were sent refrigerated to a Laboratory certified by the Ministry of Agriculture. We used infrared spectroscopy to analyze total solids, protein, fat, non-fat solids and lactose (Delta Combiscope e Bentley Combisystem B-2300) [21]. To analyze SCC [22] and BMBC we used flow cytometry (Delta Combiscope and Bentley Combisystem B-2300; Bentley Bactocount IBC) [23].

For statistical analysis, we used a log transformation of the variables BMBC and SCC to meet the conditions of normality and the mixed model (PROC MIXED, SAS version 9.0) for data analysis with repeated measures, with fixed effects for management type (C-HI, C-LI, and O-LI), season (autumn, winter, spring, summer) and their interactions. The subject of repeated measurement was the farm. Of the several error structures investigated, the heterogeneous autoregressive structure (ARH) was selected because this co-variance structure minimized the BIC (Bayes Schwarz information criterion), except for milk production data, where we used unstructured covariance (UN). For presentation purposes, data were transformed back. The effects of the management type for each season were compared using Fisher’s least significant difference. In all comparisons, significance was set at *p* ≤ 0.05. The studied variables (total farm area, percentage of area used for perennial and annual pasture, number of lactating animals per season, daily milk yield in each season, estimated daily intake of concentrate and silage per season, number of forage species consumed per season) were also submitted to principal component analysis (PCA), using the statistical package Unscrambler 9.0. Preprocessing of raw data included normalizing, and the analysis was conducted using correlation matrices with mean adjusted data.

## 3. Results and Discussion

### 3.1. Farm Characteristics

The management types differed in their productive traits (Table 1). The farms using C-HI management had the largest total area compared to the other management systems and, in general, the area with annual pasture was greater on farms with conventional management (C-HI and C-LI) compared to O-LI. The O-LI farms had the greatest area of perennial pasture. The area designated for the cultivation of perennial pasture in conventional farms (C-HI and C-LI) was only 18.8 and 23.7%, respectively, which differed significantly from O-LI farms (52.3%). In farms with C-HI and C-LI, a large proportion of the farm’s area was used for integrated crop-livestock, with a predominance of annual summer crops, mainly soybeans and corn, for grain or silage production (38.7% and 38.1% of the total area for C-HI and C-LI, respectively). In winter, these same areas were used for annual pasture cultivation. Additionally, besides having to follow environmental regulations restricting land use (20% of the farm must be protected as legal reserve area), parts of the productive area of all farms were used for the production of subsistence crops (Table 1), which is characteristic of family farming. C-HI farms had a higher number of lactating cows, with more Holsteins in their herds compared to O-LI farms, with being C-LI in an intermediary position (Table 1). Likewise, the total milk yields were 625 kg·day^−1^, 384 kg·day^−1^ and 130 kg·day^−1^ for C-HI, C-LI and O-LI farms, respectively. The primary reason for this difference is the number of lactating cows in each farm type (26.3, 19.1 and 13.1, respectively, in C-HI, C-LI and O-LI farms), but also the genetics and the feeding of the cows (Table 1 and Table 2).

**Table 1:** Characteristics of pasture-based dairy production systems in Western Santa Catarina State, Southern Brazil, grouped by conventional high input (C-HI), conventional low input (C-LI), and organic low input (O-LI) (n = 8 per group).

Items	Management Types			
	C-HI	C-LI	O-LI	SEM
Total farm area (ha)	28.9 a	21.4 a,b	18.6 b	1.75
Area of perennial pasture (%)	18.8 b	23.7 b	52.3 a	5.98
Area of annual pasture (%)	38.7 a	38.1 a	12.9 b	4.97
Other (building, crops, subsistence and legal reserve area) (%)	42.5	38.2	34.4	8.89
Breed of lactating cows (%)				
*Holstein*	50.6	33.4	20.8	3.42
*Jersey*	13.0	20.8	20.1	1.40
*Holstein Jersey crossbred*	36.4	45.8	59.1	2.99
Number of lactating animals	26.3 a	19.1 b	13.1 c	1.79
Number of lactating cows/ha	1.9 a	1.4 a,b	1.2 b	0.15

SEM = standard error of the mean. Different lower case letters on the same line indicate statistical difference between management types at a level of 5%.

### 3.2. Diet Composition, Production and Milk Quality

The use of concentrate and silage maize in conventional management occurs throughout the year (Table 2). The constant use of supplements indicates that their use was not exclusively associated with seasonal fluctuation in pasture production; they were also used as a strategy to increase milk yield (Table 3). This contrasts with the O-LI management, where the use of concentrate and/or silage was very minor, occurring only during winter and the beginning of spring. On C-HI farms the cows were left to graze during certain periods of the day, the pasture was managed rotationally and with the use of chemical fertilizers. In C-HI management, milk production relied not only on fertilized pastures, but also on the amount of concentrate and silage fed to the cows, which was greater than that used in other farm types. Thus, the average production was 22.8, 18.1 and 9.6, per cow per day in C-HI, C-LI and O-LI, respectively. The results for C-LI farms showed an intermediary situation between O-LI and C-HI. Although not relying completely on pasture, as O-LI did, they could not afford the same level of inputs as C-HI farmers, due to limited financial resources.

The nutritional management adopted by organic farmers follows Brazilian Legislation [24] in relation to the maximum (15% DM) permitted percentage of conventional supplements that must be gene manipulated organism (GMO)-free. In Brazil, GMO corn and soybean varieties dominate production, making up 90% and 92%, respectively, of all corn and soybean crops [25], and as such they are very likely grown on neighboring farms. GMO-free corn is rarely found in the region. Although some farmers cultivate their own corn, it is unlikely completely “GMO-free”, as required by law, due to the proximity to farms with GMO crops. Therefore, milk production in O-LI farms has to rely on the efficient management of pasture. To achieve a high pasture production, all O-LI farms adopted Voisin’s rotational grazing (VRG), which is an intensive and time-controlled rotational grazing system, with the aim of providing the nutritional requirements of cows while also allowing pasture regrowth. Pastures are divided into plots [26], and animals are provided with water and shade [27]. In the region, VRG farms typically have 60–80 paddocks. Milking cows stay 12 h in each paddock at an instant stocking rate of 200 cows/ha, followed by dry cows that stay in the paddock for another 12 h; the resting time of each paddock varies from 25 to 50 days, depending on the season. Subdividing large pastures may reduce distances to water and provide more uniform use of forage [28]. With a higher stocking density of animals and a shorter rotation period for each paddock, this system improves animal productivity [28,29].

**Table 2:** Estimated supplement intake for cows from farms grouped by conventional high input (C-HI), conventional low input (C-LI), and organic low input (O-LI) (n = 8 per group).

Diet Composition	Season	Management Types				
* (kg DM/cow/day)		C-HI	C-LI	O-LI	SDM	
*Concentrate*						
	Autumn	3.83	1.73	0	2.00	
	Winter	3.65	1.46	0.20	1.64	
	Spring	3.32	1.55	0.10	1.48	
	Summer	3.20	1.87	0	1.44	
*Silage*						
	Autumn	8.31	5.38	0	3.91	
	Winter	7.66	5.37	1.30	3.43	
	Spring	7.04	3.50	0.20	3.32	
	Summer	6.33	6.33	0	3.57	

* Intake of concentrate and silage was estimated by farmers and dry mass (DM) content was analyzed in concentrate and silage sampled on the farms; Statistical analyses were not performed as data available are estimates.

Despite some similarities between conventional management systems, C-LI farms appear to be intermediary between C-HI and O-LI. This is supported by similarities in the number of lactating animals, breed of animals, herd and cow milk yield and use of supplements in the diet (Table 1 and Table 2). We found that C-LI farms made limited investments in genetically improved breeds, pasture fertilization and the purchasing of large quantities of feed supplement. In relation to the identification of forage species consumed by the animals in each management type, we found that on conventional farms (C-HI and C-LI) common oats (*Avena sativa*) and annual ryegrass (*Lolium multiflorum*) were planted in autumn and winter either as a single crop or intercropped (Table 4). During the other seasons, depending on the intervals between each crop, farmers planted annual pasture in the summer, particularly Sudan grass (*Sorghum sudanensis*) and pearl millet (*Pennisetum americanum*), as a monoculture or intercropped (Table 4).

**Table 3:** Milk yield, percentage of fat, protein, lactose and total solids in milk from farms grouped by conventional high input (C-HI), conventional low input (C-LI), and organic low input (O-LI) (n = 8 per group) in Western Santa Catarina State, Southern Brazil.

Parameters	Season	Management Types			
		CH-I	CL-I	O-LI	SEM
Milk yield (kg/lactating cow/day) corrected fat 4%	Autumn	23.1 ABa	16.8 b	9.2 c	1.45
	Winter	22.1 Ba	18.0 ab	10.8 c	1.45
	Spring	25.8 Aa	19.2 ab	9.8 c	1.45
	Summer	22.8 ABa	19.3 ab	10.2 c	1.45
Fat (%)	Autumn	4.4 Aa	4.1 Aa	4.4 Ba	0.20
	Winter	4.0 ABa	3.9 Aa	4.8 Ab	0.21
	Spring	4.5 Aa	4.2 Aa	4.0 Ba	0.22
	Summer	3.9 Ba	4.1 Aa	4.1 Ba	0.15
Protein (%)	Autumn	3.4	3.2	3.3	0.10
	Winter	3.7	3.3	3.3	0.25
	Spring	3.2	3.3	3.2	0.07
	Summer	3.1	3.3	3.1	0.06
Lactose (%)	Autumn	4.3	4.3	4.3	0.09
	Winter	4.5	4.4	4.4	0.07
	Spring	4.5	4.4	4.6	0.05
	Summer	4.4	4.4	4.6	0.07
Total Solids (%)	Autumn	13.1 Aa	12.6 Aa	12.9 Aa	0.26
	Winter	11.9 Ba	11.8 Ba	12.6 Ab	0.21
	Spring	13.2 Aa	12.9 Aa	12.7 Aa	0.21
	Summer	12.3 Ba	12.6 Aa	12.5 Aa	0.18

SEM = standard error means between management types. Different lower case letters in the same row indicate statistical differences between management types in the same season; different upper case letters in the same column indicate statistical differences of the same management types between seasons (*p* < 0.05).

Higher levels of fat in organic milk in comparison to conventional milk have also been attributed to the predominant use of Jersey cows, animals in the early stages of lactation and a greater consumption of fresh forage on organic farms [10]. In our study, however, we controlled for the effect of breed and lactation stage by excluding animals that did not fit within the determined breed type (Holstein x Jersey) and animals that were between the 4th and 6th month of lactation.

Pasture management on VRG can explain the greater species richness encountered on the organic farms, and consequently, the greater consumption by the animals (Table 4). Previous studies have demonstrated an increase in the diversity of species through pasture management in comparison to unmanaged fields [30], as well as the positive effect of intensive management on pasture quality. In this case, the pasture achieves higher levels of protein and lower crude fiber than extensively managed pasture [30]. Adler *et al.* [13] also found differences in botanical composition, with a lower proportion of grasses and greater proportion of legumes on organic farms compared to conventional farms. C-HI and C-LI farmers managed their perennial pastures on a rotational system with fixed resting days, where the cows stayed on pasture for part of the day and in the barn receiving silage and concentrate for the other part of the day. Weeds are controlled with chemicals, and soluble fertilizers are used on the pasture. None of these methods are used on the O-LI farms. The management methods used may explain the absence of legumes found in the cows’ diet on C-HI and C-LI farms.

**Table 4:** Forage species identified in simulated grazing samples on two or more farms grouped by conventional high input (C-HI), conventional low input (C-LI), and organic low input (O-LI) (n = 8 per group) in Southern Brazil.

Season	Management Types	Plant Species		
		Cultivated Grass	Legume	Native Forage
Autumn	C-HI	*Avena sativa*;		*Urochloa plantaginea*
		*Eleusine indica*;		
		*Sorghum sudanensis*		
	C-LI	*Avena sativa*; *Avena strigosa*; *Lolium multiflorum*; *Sorghum sudanensis*		*Cynodon dactylon*
	O-LI	*Axonopus affinis*; *Axonopus campressus*; *Lolium multiflorum*; *Saccharum officinarum*; *Pennisetum purpureum*	*Trifolium repens*	*Ageratum conyzoides*; *Amaranthus deflexus*; *Amaranthus spinosus*; *Kylling abrevifolia*; *Paspalum mandiocanum*; *Paspalum umbrosum*; *Paspalum urvillei*; *Plantago australis*; *Solidago chilensis*
Winter	C-HI	*Avena strigosa*; *Lolium multiflorum*		
	C-LI	*Avena strigosa*; *Lolium multiflorum*		
	O-LI	*Avena strigosa*; *Lolium multiflorum*	*Trifolium repens*	*Bowlesia incana*; *Bromus catharticus*; *Cerastium glomeratum*; *Hypochaeris megapotamica*; *Hypochaeris* sp.; *Paspalum umbrosum*; *Plantago australis*; *Veronica arvensis*
Spring	C-HI	*Avena sativa*; *Lolium multiflorum*		
	C-LI	*Avena sativa*; *Lolium multiflorum*		
	O-LI	*Avena strigosa*; *Lolium multiflorum*	*Trifolium repens*; *Vicia sativa*	*Cynodon defensis*; *Leonurus sibiricus*; *Paspalum urvillei*; *Penisetum setaceum*; *Plantago australis*
Summer	C-HI	*Pennisetum americanum*; *Sorghum sudanensis*		
	C-LI	*Pennisetum americanum*; *Sorghum sudanensis*		
	O-LI	*Pennisetum purpureum*; *Sorghum sudanensis*	*Desmodium affine*; *Glicine max*	*Digitaria ciliaris*; *Euphorbia heterophylla*; *Paspalum juergensii*; *Paspalum umbrosum*; *Urochloa plantaginea*

Data obtained based on the methodology described by Euclides *et al.* [20].

We found an inverse correlation between the percentage of the farm area used for annual pasture and lower levels of pasture richness (r^2^ = −0.70; *p* < 0.05), as well as a positive correlation between the percentage of land used for perennial pasture and species-rich pasture (r^2^ = 0.74, *p* < 0.05). As can be seen in Table 1, O-LI farms have significantly more perennial pasture than C-HI and C-LI, with greater species richness, which is an indicator of better sustainability and higher productivity [31]. Perennial pasture is related to improved sustainability and productivity because their long-lived deep root systems take up soil water more efficiently, reduce soil acidification and reduce the risk of soil erosion by maintaining plant cover during summer, improve soil structure and water infiltration, and provide feed for livestock when annual pastures have senesced [32]. Pastures made up of only one or two species often have less production stability than pastures with a greater complexity of species [33]. In this sense, increasing the pasture diversity may represent an important strategy for the maintenance of pasture-based animals. Mixed pastures can be more profitable and more productive than monocultures, while also providing less risk of weather-related loss than pastures made up of only one grass species [34].

The milk yield per cow per day was greater in the C-HI farms compared to organic farms in every season throughout the year (Table 3). The lower milk yield per cow seen in O-LI farms is likely associated with the use of mixed breed animals and a diet with low input of supplements, along with smaller farm size and lower number of lactating cows. These characteristics explain the lower milk yield at the level of the herd, which seems to be something that could be improved on organic farms. Previous studies have shown that a large proportion of organic [7,35] and low-input conventional farms (that make limited use of external supplements) tend to breed crosses that provide more hardy animals that are better adapted to local climatic conditions such as temperature and more suitable to hilly terrains typical of the region [4,10,16]. In general, despite lower milk production, farmers have adopted crossbreeds to give more emphasis on traits such as fiber use efficiency, fertility, health and survival in the herd [36].

Higher levels of fat and total solids were found in organic milk in the winter compared to the other systems. The literature shows that fat is the milk component that can present the greatest variation, and diet is responsible for 50% of this variation [37]. It is important to note that the current study used cows in similar lactation stages in the three studied management systems, and that O-LI cows received very little concentrate (Table 2). Therefore, the greatest levels of fat and total solids present in organic milk in the winter are likely due to pasture quality and diet, as seen in previous studies [38,39]. Despite differences in nutritional management shown in Table 2 and Table 4, no other difference was found in the quality of milk. Levels of protein and lactose in the milk averaged 3.28% ± 0.08% and 4.41% ± 0.05%, respectively, and did not vary between management types or across seasons (Table 3).

In relation to seasonality, the level of fat was lower in summer only for C-HI and higher in the winter for O-LI. We found no further significant differences. The lower fat for C-HI could be considered unexpected due to the higher fiber content in pasture occurring in this season [40]. C-HI farms had a higher proportion of crops that occur mostly in the summer. Therefore, in the summer we can expect a lower pasture area for these farms, resulting in an increased speed of rotation on their pasture, composed only of highly digestible annual species (Table 4). It was not possible to analyze the bromatological composition of the diet. Therefore, we can only infer that the summer pastures were grazed too early, when pasture composition was likely very low in fiber. Regarding the higher fat content in the milk from O-LI farms in the winter, we can only infer that the opposite occurred. Although the cows were fed ryegrass, oats and clover, three highly digestible species, there was also a high prevalence of tropical species in the diet, which would provide high levels of fiber. In fact, in our analysis we observed the cows eating these species (Table 4).

Considering the hygienic quality of milk, no significant differences were found between management types and seasons for BMBC or BTSCC (Table 5). Overall, the values found for BTSCC are within the limits allowed by Brazilian legislation, but the maximum values for BMBC found are alarming. At the time of the study, Brazilian legislation allowed a maximum value for both BTSCC and BMBC of 600,000 per mL, and the results demonstrate the inability of many farms to comply with existing regulations. Regarding milking conditions in the farms studied, all used milking machines, but on the majority of farms (15 out of 24) the milk was collected in a receiving jar and manually transferred to a cooling tank. On five farms, the milk collected was kept in the jars, which was placed inside cool water in cooling tanks. Only on five farms—four C-HI and one C-LI—was a pipeline system in place to transport the milk to the cooling tank. Excessive manipulation of the milk, such as the manual transfer from one recipient to another, and delay in the transfer to the cooling tank may influence contamination and growth of bacteria [41]. The dairy company collected milk on average every second day (range 1–4 days), which could influence high BMBC in general. Nonetheless, we found no differences among management types. We could not identify any influence of the type of equipment or milking parlor environment on BMBC data. Because of the paramount importance of this information for human health, further studies must be done to identify the causes of high BMBC in order to develop strategies to mitigate against such high bacteria counts.

The main factors contributing to increased BMBC in milk are post-harvest and related to environment and hygiene: ambient temperature, accumulation of mud due to periods of rain resulting in unclean teats, contaminated facilities, improper management and poor cleaning of milking machines, inadequate refrigeration of milk, and contact of milk with air, among others [42]. Moreover, bacterial contamination of raw milk can generally occur from three main sources: within the udder, outside the udder (cow environment) and the surface of milk handling and storage equipment. The degree of cleanliness of the milking system is a major factor influencing the total bulk milk bacteria count [41]. Considering the low risk of the cows’ environment in the present study (cows spent most of the time on pasture), the high BMBC is probably due to a lack of cleaning and sanitizing procedures in milking machines and storage equipment and the high BTSCC due to high levels of mastitis.

**Table 5:** Bulk Milk Bacteria Count (BMBC)* and Bulk Tank Somatic Cell Count (BTSCC) in milk collected from cooling tanks on the farms grouped by conventional high input (C-HI), conventional low input (C-LI), and organic low input (O-LI) (n = 8 per group) management (n = 8 per group).

Season	Management Types	BMBC (×1000 CFU/mL)	SEM	BTSCC (×1000 cells/mL)	SEM
Autumn	C-HI	1226	724.4	775	231.7
	C-LI	1408	724.4	545	231.7
	O-LI	1472	724.4	609	231.7
Winter	C-HI	223	958.4	498	113.4
	C-LI	2174	958.4	469	113.4
	O-LI	498	958.4	350	113.4
Spring	C-HI	418	197.4	301	85.6
	C-LI	475	197.4	575	85.6
	O-LI	801	197.4	297	85.6
Summer	C-HI	444	131.6	353	97.4
	C-LI	557	131.6	467	97.4
	O-LI	393	131.6	308	97.4

* Mean values represented. SEM = standard error of the mean.

Comparing the standards established by the principal milk-producing countries worldwide for BTSCC and BMBC, we can see some differences among them. The majority of European countries, New Zealand and Australia have maximum limits of BTSCC of 400,000, in Canada the limit is 500,000 and in the USA the limit is 750,000 cells/mL [43]. In the USA, there have been attempts to reduce this limit to 400,000 cells/mL, but the National Conference on Interstate Milk Shipments [44] did not find a hygiene-related justification for such a modification. The maximum BMBC permitted for all countries noted above is 100,000 CFU/mL. Regardless of the management type used, we found higher values than those permitted by the Brazilian legislation at the time of the study [24] on a significant number of farms primarily for BMBC. As of July 2016, Brazilian law will require these values to be below 400,000 cells/mL for BTSCC and 100,000 CFU/mL for BMBC, which presents a major challenge for the dairy industry. In order to adapt to these new regulations, farmers must adopt drastic measures to control mastitis and hygiene during and after milking.

**Figure 1:**
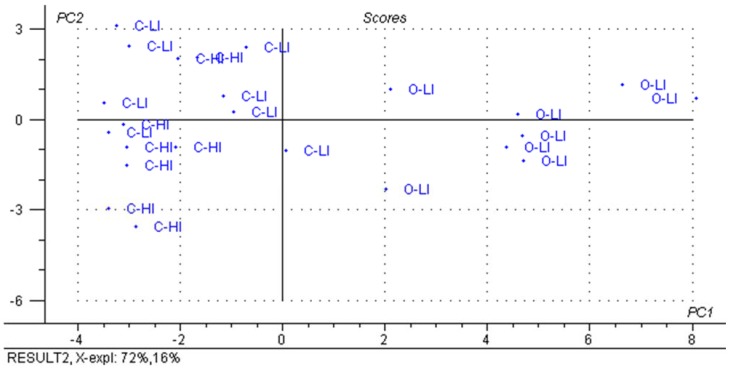
Factorial distribution of principal component 1 (PC1) and principal component 2 (PC2) for all studied descriptors of farms grouped by conventional high input (C-HI), conventional low input (C-LI) and organic low input (O-LI) (n = 8 per group).

**Figure 2:**
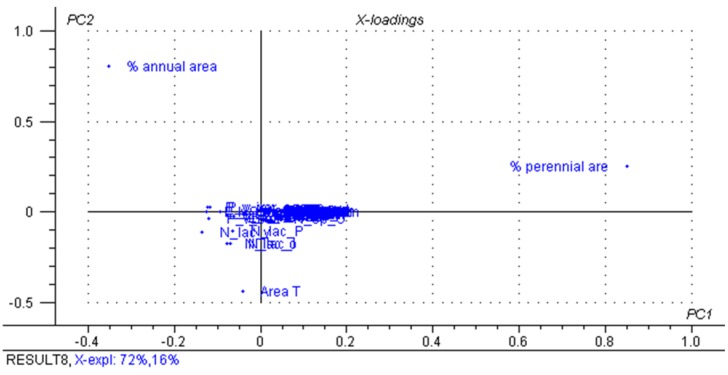
Factorial contribution of principal component 1 (PC1) and principal component 2 (PC2) for the classification of farms based on all descriptors: total farm area, % of area annual pasture, % of area perennial pasture, average number of forage species, number of species per season, intake of concentrate and silage per day and cow in each season, % of fat, protein, lactose, total solid, non-fat dry extract, BTSCC and BMBC values.

From the distribution of the studied variables in the total data set matrix, we can see a clear separation between organic farms and the conventional farms in axis PC1, which explains 76% of the variability in the data (Figure 1). The O-LI farms are located in PC1+ and the conventional farms in PC1-, with no distinction between farms managed by C-HI or C-LI (Figure 1). The descriptors that contribute to a classification of samples in PC1 and PC2 are the percentages of total farm area designated for perennial and annual pasture, respectively (Figure 2). In this case, the land use was the factor responsible for the grouping (Figure 2). While other attributes initially considered important for the distinction between groups were included in the analysis, such as the use of supplements in cow diet, the results showed little significance. In previous studies, PCA was able to demonstrate a distinction of milk samples based on the region or management system used [11,45].

Above, we mentioned the results of the PCA analysis when noting that on average half of the area of organic farms was designated for the cultivation of perennial pasture, representing almost double that used in conventional management (Table 1). The extent of this finding goes beyond the specific differences in land use, given that the perennial vegetation more effectively provides essential benefits for eco-efficiency of the management system. Previous studies show that annual crops result in significant reductions in soil fertility and require intensive inputs to maintain productivity, while perennial pastures maintain high levels of soil fertility as well as more complex biological communities [31,46]. Furthermore, in terms of the trophic chain, perennial pasture promotes a greater number and diversity of pollinating insects, herbivores and decomposers [47]. As such, perennial crops are a valuable ecological resource for sustainable agriculture.

## 4. Conclusions

The three pasture-based systems differed in type of pasture (perennial or annual), farm size, intake of concentrate and silage and pasture diet. The O-LI had the smallest farms and herds, lowest input and lowest milk yields compared to C-HI and C-LI. C-HI had higher inputs and milk yields than C-LI. Total milk composition was little affected by production system and season, but BTSCC was generally high and BMBC exceeded the acceptable level in all systems and all seasons. Based on the available data we may conclude that C-HI and O-LI systems are economically more beneficial for the farmer; however, the low milk yields on O-LI farms are a major concern. Besides milk production, O-LI farms may contribute to important eco-system services. Cell count and hygienic quality need to be addressed in all production systems especially during the autumn.
